# Reimagining U.S. social care policy: a commentary on Medicaid strategies to better support individuals with dementia

**DOI:** 10.3389/frdem.2026.1820253

**Published:** 2026-04-16

**Authors:** Alice Boughrum, Amy Lastuka, Nicole Batsch, Victoria Helmly, Mary Manchester, Lillian Morgado, Romil R. Parikh, Mary Louise Pomeroy, Audrey Price, Michael Splaine, Raven H. Weaver, William Zagorski, Regina A. Shih

**Affiliations:** 1 Georgia Health Policy Center, Georgia State University, Atlanta, GA, United States; 2 USA Institute for Health Metrics and Evaluation, University of Washington, Seattle, WA, United States; 3 Community Partner, Florida, FL, United States; 4 School of Public Health, Georgia State University, Atlanta, GA, United States; 5 Division of Health Policy and Management, University of Minnesota School of Public Health, Minneapolis, MN, United States; 6 Center for Equity in Aging, Johns Hopkins School of Nursing, Baltimore, MD, United States; 7 Owner Splaine Consulting, Managing Partner, Recruitment Partners LLC, Columbia, SC, United States; 8 Department of Human Development, Washington State University, Pullman, WA, United States; 9 American Senior Care Centers Inc, Nashville, TN, United States; 10 National Adult Day Services Association, Fairfax, VA, United States; 11 Emory University Rollins School of Public Health, Atlanta, GA, United States; 12 RAND Arlington, Arlington, VA, United States

**Keywords:** dementia, Long-Term Services and Supports, Medicaid home and community-based services, Medicaid, state policy

## Introduction

An estimated 7.2 million Americans 65 and older live with dementia, a number expected to double over the next 50 years (Alzheimer's Association, 2025). More than 80% of people living with dementia (PLWD) live in the community, relying on support from family and friends and home and community-based services (HCBS) to delay or avoid institutional care (Harris-Kojetin et al., 2013; Lepore et al., 2017; Knapp et al., 2016; Olsen et al., 2016; Binette and Farago, 2021; Wehrmann et al., 2021). HCBS encompass a wide range of medical and social services, including food and nutrition programs, personal support services, adult day services, transportation, family caregiver support, and respite care, among other services (Casado and Lee, 2012; Konetzka et al., 2020; Wang et al., 2021; Mann et al., 2023).

HCBS are particularly critical for PLWD, who differ in important ways from other HCBS consumers. PLWD experience a progressive decline in memory and other cognitive functions, which leads to impairment in higher-level skills and everyday tasks like basic self-care and the need for verbal or written cues and monitoring to complete daily activities (Raimo et al., 2024). Disease progression is also accompanied by complex medical and behavioral health needs (Cerejeira et al., 2012; Alpert and Fain, 2025). Consequently, PLWD rely to a greater extent on support to remain in the community compared to people with many other types of disabilities. Despite these needs, HCBS access and quality for PLWD vary considerably across the U.S., and identifying state-level policy solutions is essential for improving outcomes among PLWD.

Medicaid, a joint federal and state program, is particularly salient to HCBS policy discussions, as it serves as the primary payer for HCBS in the U.S., providing support to 4.5 million Americans (Mohamed et al., 2025). However, access to Medicaid HCBS is uneven, and PLWD experience unique barriers to receiving these services. Unlike institutional care, which is covered for eligible individuals by Medicare for short-term stays and covered by Medicaid for longer-term stays, nearly all HCBS are optional benefits under Medicaid (Burns et al., 2024; Mohamed et al., 2025). States are only required to cover a subset of HCBS categorized as home health services, which include part-time nursing services, home health aide services, and medical supplies, equipment, and appliances, and decisions around covering other HCBS through Medicaid are left to the discretion of states (Mohamed et al., 2025). The discretionary nature of most Medicaid HCBS alongside a host of factors, namely the complex structure of most state Medicaid HCBS programs, state-by-state variation in Medicaid policies, and states' eligibility HCBS determination processes, have resulted in inconsistent and often inequitable access to HCBS for many PLWD (Rohde et al., 2024).

Recent research highlights important barriers to HCBS for PLWD, suggesting that the HCBS system is not dementia-capable. As defined by the Administration for Community Living, a dementia-capable system acknowledges that PLWD need more and different services than people with physical disabilities and are more likely to rely on caregivers (Tilly et al., 2014). Among these barriers difficulties obtaining a diagnosis—which may negatively influence eligibility for comprehensive HCBS—as well as limited awareness of available services and fear of stigmatization emerge as especially prominent (Waymouth et al., 2023). (Shippee et al. 2025) report that, among existing HCBS consumers, a dementia diagnosis is associated with a higher likelihood of reporting unmet HCBS needs despite higher HCBS utilization among PLWD. Given that most PLWD prefer to remain in the community and often cannot afford to pay out of pocket for in-home or institutional care (Reckrey et al., 2020), examining challenges and identifying pathways to improve Medicaid-funded HCBS access is critical to promoting equitable, person-centered dementia care and preventing unnecessary institutionalization.

This commentary aims to link critical gaps in Medicaid-funded HCBS access for PLWD in existing research and identify policy opportunities to address current access issues. We argue that the most pressing priorities are to leverage flexible waiver authorities, revise eligibility determination processes, and implement reforms that promote equity and transparency around eligibility determination decisions.

### Medicaid waivers: a promising tool for improving access to HCBS for PLWD

States have two main pathways for offering Medicaid HCBS: state plan benefits and waivers (Rohde et al., 2024). HCBS offered by state plans must be available to all eligible individuals within a state, and states are not generally allowed to limit services within specific geographic boundaries, set enrollment caps, or establish waiting lists (Rohde et al., 2024). In contrast, states can offer HCBS through waivers, which allow for limiting benefits to certain groups based on geography, income, or disability (Mohamed et al., 2025). Given the substantial latitude they offer, most states choose to provide HCBS through waivers, using them to establish specific eligibility standards, scope of services, and delivery models for their programs. Though this flexibility comes with its aforementioned shortcomings, it also affords states opportunities to design waivers with attention to certain populations and target services to better meet consumers' needs and preferences (Haltermann et al., 2023; Miller et al., 2025). Waivers can also serve as a unique strategy for addressing social determinants of health and reaching consumers earlier in their disease progression, potentially improving health outcomes and alleviating financial strain on consumers and families, as well as states (Lipson, 2017; Medicaid and CHIP Payment and Access Commission, 2022). These features make Medicaid waivers a powerful policy tool states can leverage to meet the complex medical and social needs of PLWD, who comprise a large and growing share of their populations.

### State policy opportunities

In their design of HCBS waivers, states can take proactive steps to increase access to and improve the quality of Medicaid HCBS to meet the needs of PLWD. These actions include expanding eligibility through flexible Medicaid authorities, revising aspects of the eligibility determination process, and ensuring equity and transparency around eligibility determinations.

#### Using flexible waiver authorities to expand access

Waivers offer states opportunities to modify eligibility rules to increase access to HCBS for specific groups and broaden service offerings to target particular needs. Several states have used waiver flexibilities to pilot programs targeting HCBS to people before they meet nursing home-level of care criteria, which is an eligibility requirement for most waivers. Vermont, for example, provides HCBS to individuals aged 18 and older who are at risk of institutionalization but do not yet meet a nursing home level of care through its 1115(a) Global Commitment to Health Waiver, Vermont's Choices for Care (CHC). The program provides supports for individuals with “moderate needs,” including adult day and homemaker services and flexible funding that can be used to employ a family member or friend as an “attendant” (Vermont Department of Disabilities, Aging and Independent Living, 2016). Other 1115 demonstrations, such as New Jersey's Family Care Comprehensive Demonstration, include housing and social supports for high-risk populations, such as tenancy and transition supports or short-term services aimed at preventing or delaying institutionalization (Hinton and Diana, 2024). In addition to 1115 demonstrations, several states have adopted Community First Choice [1915(k)] or expanded state-plan HCBS to broaden access to attendant and support services that can stabilize people in the community (e.g., North Carolina Division of Health Benefits, 2023). States were also recently granted the authority to apply for new 1915(c) HCBS waivers under Pub. L. No. 119-21, 139 Stat. 317 (2025), which allows states to provide Medicaid HCBS to individuals without requiring them to meet an institutional level of care, providing it does not increase any waiting time. Although it will not go into effect until 2028, this pathway could allow for earlier intervention in targeted populations, including PLWD to prevent institutionalization while balancing state budget constraints under traditional 1915(c) waiver authority.

These examples illustrate the flexibility waivers offer states in designing their eligibility criteria and service array. However, while these and similar waivers expand access to HCBS for many consumers, few states have used flexible waiver authorities to explicitly target populations with cognitive impairment outside of specific conditions such as traumatic brain injuries. In their eligibility criteria, states often prioritize functional over cognitive impairments, or do not consider cognitive impairments at all, potentially excluding PLWD from services (Alzheimer's Association, 2025). While using more moderate-level needs criteria can improve access for some individuals with dementia, states need to clearly prioritize cognitive, rather than physical, impairments in their eligibility rules to ensure PLWD with lower levels of physical disability can access services. States should also consider eligibility assessments that better evaluate needs among PLWD and tailor waiver services accordingly, potentially increasing the quality and effectiveness of available HCBS for PLWD. An approach that could more fully account for PLWD's needs could involve including family caregivers' needs in the assessment process when a caregiver is available, as the care plans for PLWD are often dependent on family caregivers. This approach warrants further exploration and, if applied, states should thoughtfully consider what caregiver assessments to include and how they are used in the eligibility process, as current assessment practices vary widely by state and local agency, and this strategy will not be an option for PLWD without a care partner (Shugrue et al., 2019).

#### Revising eligibility determination processes

The Medicaid waiver application process is a major barrier to HCBS for PLWD. In many states, eligibility includes being age 60 or older or having a documented disability. Individuals seeking coverage through the disability category must also receive a disability determination through the Social Security Administration (SSA) or a state agency (Colello and Morton, 2019). Applicants face additional barriers to obtaining disability status if they do not have a formal diagnosis, which is required for the disability evaluation process through the SSA (Waymouth et al., 2023; Social Security Administration, 2026). Underdiagnosis of dementia is a widespread problem in the U.S., especially among minoritized racial and ethnic groups, and studies suggest that less than half of PLWD have been diagnosed by a clinician (Cox et al., 2025). Obtaining a formal dementia diagnosis is often a lengthy process complicated by a number of factors, and individuals are frequently misdiagnosed, especially those with rarer subtypes of dementia (Lai et al., 2023; Giebel et al., 2024). As an alternative to requiring a formal diagnosis to meet eligibility criteria for HCBS, states can encourage but not require individuals to obtain a diagnosis for waiver eligibility. This modification could remove a significant barrier to HCBS access for PLWD.

Another option states can leverage to reduce this barrier to accessing HCBS is to implement presumptive eligibility policies. Presumptive eligibility allows states to use basic financial information and screening tools to grant temporary eligibility while an individual waits for their full application to be processed (Reinhard et al., 2021). Several states have adopted presumptive eligibility policies for Medicaid HCBS, including Washington state under its 1115 Medicaid Transformation Project waiver. Washington's program established presumptive eligibility for individuals who need HCBS through Medicaid state plan and 1915(c) waiver authorities, and the process involves a brief screening by Area Agency on Aging staff to determine and grant presumptive eligibility, facilitating access to services while the individual awaits a final eligibility determination (Washington State Health Care Authority, 2025).

#### Ensuring equity and transparency

Certain subgroups of PLWD may also experience persistent disparities in HCBS access, particularly those living in rural areas. Multiple factors are thought to contribute to these disparities, including PLWD in rural areas facing greater barriers to care due to stigma, cultural beliefs, and limited information about dementia (Waymouth et al., 2023). Direct care workforce shortages also disproportionately affect rural areas, and recent research has found that the ratio of direct care workers to older adults is substantially lower in rural compared to urban areas of the U.S. (Dill et al., 2023). These gaps in access are especially concerning in light of research that suggests people in rural areas have a higher risk of dementia and shorter survival times compared to their urban counterparts, and that rural areas are experiencing more rapid population aging, potentially further exacerbating existing disparities (Cohen and Greaney, 2023; Kramer et al., 2025).

Recognizing the myriad barriers to HCBS access in rural areas, several states have implemented structured family caregiving services to increase support options amid workforce shortages. Through structured family caregiving, state Medicaid programs provide payment and support, including training and respite, to family caregivers who, in turn, provide personal care to the Medicaid beneficiaries, rather than beneficiaries receiving traditional services from a direct care worker (Kaye, 2022). Missouri specifically targets PLWD under its 1915(c) Structured Family Caregiving waiver, limiting eligibility to those with a dementia diagnosis (Medicaid Planning Assistance, 2025). Although Missouri's requirement to obtain a formal diagnosis may bar access for some PLWD and structured family caregiving may not be a viable option for PLWD who have limited support networks more broadly, the service holds promise for addressing some gaps in care for PLWD in rural areas. In addition to structured family caregiving, many states are using newly awarded Rural Health Transformation Program (RHTP) funds toward initiatives aiming to strengthen HCBS access in rural areas, including growing the rural direct care workforce, supporting caregivers, and enhancing care coordination, and several states have dementia-focused initiatives (Centers for Medicare and Medicaid Services, n.d.; Taggart, 2026). Though the extent to which these initiatives are successful and implemented as intended will take time to emerge, the RHTP presents promising opportunities for investment in the rural HCBS infrastructure.

Another critical issue related to equity is transparency around waiver policies and eligibility determination decisions. Policies and other publicly available information about waiver programs are often unclear about how applicants are prioritized for services, including those on waiting lists. According to a 2024 report, individuals who were placed on waiting lists for HCBS waivers waited an average of 40 months before receiving services (Burns et al., 2024). Lack of awareness and knowledge about Medicaid waiver services among PLWD and their families is a pervasive barrier to HCBS access (Scattarelli et al., 2026). While there are efforts at the federal level to streamline Medicaid eligibility and enrollment processes, states have an opportunity to make concerted efforts to increase the transparency of these processes to help PLWD and their families access the information they need to navigate their available options.

### Enabling evidence-based HCBS state policy evaluation

Underlying these recommendations is a need for research that connects state policies to outcomes for PLWD, which has been impeded by inadequate and inconsistent data. Increased transparency and reporting from states would open up opportunities for rigorous analysis of the costs and benefits of HCBS policy decisions. States should seek to provide standard reporting metrics, such as time to institutionalization, functional trajectories, and consumer and caregiver-reported quality outcomes. Additionally, states should provide details about policy implementation, particularly around eligibility determination decisions, including what screening instruments are used, how their results are interpreted, who screens applicants, how applicants are prioritized for services, and who makes final decisions about eligibility. Several federal efforts, including the Ensuring Access to Medicaid Services Final Rule (the “Access rule”) and Guiding an Improved Dementia Experience (GUIDE) Model, may lay the groundwork for this research through their requirements around systematic data collection, use of uniform metrics, and consistent reporting (Centers for Medicare and Medicaid Services, 2024, 2026). This additional transparency would allow for systematic assessments of policy impacts on access to and use of Medicaid HCBS among PLWD, both within and across states and HCBS delivery systems.

## Limitations

These recommendations have clear potential benefits for multiple stakeholders. For instance, by mitigating barriers to HCBS utilization, states can potentially reduce expenditures on institutional care while also improving quality of life and cost of living for Medicaid enrollees and their informal caregivers. The policy opportunities highlighted in this commentary can make this possible. However, any recommendation to expand access to HCBS also comes with inherent challenges. States have finite budgets and are operating in a tightening fiscal landscape and, importantly, workforce capacity and provider readiness present persistent roadblocks to HCBS access. The HCBS workforce has not grown to meet the demand for services. These shortfalls are expected to worsen over time (Kreider and Werner, 2023) and may add additional pressure on informal caregivers or PLWD living alone. Disadvantages of waivers include their complexity, time-limited nature, and budget-neutrality constraints, which can limit scale. Unfortunately, there is limited rigorous evidence on the trade-offs involved in expanding or contracting HCBS access, including long-term cost offsets. Additionally, which combination of services may most effectively prevent or delay nursing home entry is difficult to measure given priorities for individualized, person-centered care plans. There are also policy opportunities, such as those possible through managed care models, that we did not explore in this commentary. States with Managed Long-Term Services and Supports programs can incorporate many of these considerations around program eligibility and tailoring services for PLWD in addition to other tools to strengthen HCBS for PLWD.

## Conclusion

HCBS improve the lives of many individuals who wish to age in the community. However, gaining access to these services can be difficult, and the barriers are compounded for PLWD. States have promising opportunities to strengthen the HCBS system of care for PLWD through the use of flexible waiver authorities and designing policies to better account for and address the needs of PLWD.

As states take steps to improve Medicaid HCBS, both CMS and states can consider opportunities to support research on HCBS. Recent CMS initiatives like the Access rule may facilitate this work, which has thus far been limited by a lack of data resulting from the inconsistent implementation of evidence-based tools, standardization of metrics, and state-by-state policy variation. Ongoing research is critical to understand the extent to which PLWD can access HCBS and the outcomes of HCBS utilization. This information will allow states to learn from each other and redesign programs to better meet Medicaid enrollees' needs, improve access, and allocate HCBS budgets efficiently and equitably.
